# Receiver Operating Characteristic Curve Analysis May be Helpful to Study the Prognostic Value of miR-155 in B-Cell Non-Hodgkin Lymphoma

**DOI:** 10.4274/tjh.2017.0297

**Published:** 2017-12-01

**Authors:** Long Su

**Affiliations:** 1 The First Hospital of Jilin University, Department of Hematology, Changchun, China

**Keywords:** Non-Hodgkin lymphoma, miR-155, Prognostic value

## To The Editor,

Aberrant expression of microRNA (miR-155) has been reported previously in several hematological malignancies [1,2,3,4]. Recently, Bedewy et al. [5] published their excellent findings in this journal. They reported that miR-155 expression was significantly upregulated in patients with B-cell non-Hodgkin lymphoma (NHL) compared with normal controls. In patients with B-cell NHL, a high level of miR-155 expression was associated with the presence of B symptoms, involvement of extranodal sites, and high Eastern Cooperative Oncology Group score. In patients with diffuse large B-cell lymphoma (DLBCL), high miR-155 levels were related to non-germinal B-cell-like type and higher International Prognostic Index scores. High miR-155 expression was also associated with inferior event-free survival. Accordingly, the authors concluded that miRNA-155 might be a potential biomarker of prognosis and monitoring in B-cell NHL, and especially that of the DLBCL type [5].

Receiver operating characteristic (ROC) curve analysis is a graphical plot that illustrates the diagnostic ability of a binary classifier system as its discrimination threshold is varied. The best cutoff value can be calculated by ROC analysis for continuous variables to predict dichotomous variables with the best sensitivity and specificity. In Bedewy et al.’s [5] study, the patients were divided into high-expression and low-expression groups based on the median level of miR-155 relative expression units. I wonder if the median value of miR-155 expression level in this study was the cutoff value with the best sensitivity and specificity between patients and normal controls by ROC analysis. Similarly, in patients with B-cell NHL, whether there is another cutoff value that is associated with clinical stage, treatment response, long-term outcomes, and so on remains to be determined.
